# Spatio-Temporal Modification of Lignin Biosynthesis in Plants: A Promising Strategy for Lignocellulose Improvement and Lignin Valorization

**DOI:** 10.3389/fbioe.2022.917459

**Published:** 2022-07-01

**Authors:** Yongli Wang, Cunjin Gui, Jiangyan Wu, Xing Gao, Ting Huang, Fengjie Cui, Huan Liu, Sivasamy Sethupathy

**Affiliations:** ^1^ Biofuels Institute, School of the Environment and Safety Engineering, Jiangsu University, Zhenjiang, China; ^2^ School of Food and Biological Engineering, Jiangsu University, Zhenjiang, China

**Keywords:** lignin biosynthesis, lignin valorization, genetic modification, spatio-temporal, promoter

## Abstract

Lignin is essential for plant growth, structural integrity, biotic/abiotic stress resistance, and water transport. Besides, lignin constitutes 10–30% of lignocellulosic biomass and is difficult to utilize for biofuel production. Over the past few decades, extensive research has uncovered numerous metabolic pathways and genes involved in lignin biosynthesis, several of which have been highlighted as the primary targets for genetic manipulation. However, direct manipulation of lignin biosynthesis is often associated with unexpected abnormalities in plant growth and development for unknown causes, thus limiting the usefulness of genetic engineering for biomass production and utilization. Recent advances in understanding the complex regulatory mechanisms of lignin biosynthesis have revealed new avenues for spatial and temporal modification of lignin in lignocellulosic plants that avoid growth abnormalities. This review explores recent work on utilizing specific transcriptional regulators to modify lignin biosynthesis at both tissue and cellular levels, focusing on using specific promoters paired with functional or regulatory genes to precisely control lignin synthesis and achieve biomass production with desired properties. Further advances in designing more appropriate promoters and other regulators will increase our capacity to modulate lignin content and structure in plants, thus setting the stage for high-value utilization of lignin in the future.

## Introduction

Lignocellulosic biomass, which comprises polysaccharides (cellulose and hemicelluloses) and lignin, is the most abundant renewable resource provided by plants (Iqbal et al., 2013). Because of its renewable nature, lignocellulosic biomass holds great promise for a sustainable bioeconomy, as it can be utilized in the production of biofuels, enzymes, chemicals, paper, animal feed, and composites. Lignin occurs as a monomer or a polymer, and is the second most abundant polymer after cellulose, accounting for 10–30% of the biomass of most plants (Schutyser et al., 2018). Guaiacyl (G), p-hydroxyphenyl (H) and syringyl (S) units are the major building blocks of lignin and are derived from monolignols including ρ-coumaryl alcohol, coniferyl alcohol, and sinapyl alcohol, respectively. Phenyl group and a propyl side chain are present in the structure of G, H, and S units, thus they are commonly known as phenylpropane units. In lignin, these phenylpropane units are linked through an β-aryl ether (β-O-4), α- aryl ether [(α-O-4) cyclic and non-cyclic], diaryl ether (5-O-4), aliphatic ether (5-5′, α-O-γ), resinol (β-β), spirodienone (β-1), phenylcoumaran (β-5, cyclic β-5), biphenyl (5-5′), 8-β, and 8-8 linkages (Li et al., 2015). Hydroxyl (phenyl, benzyl and aliphatic), methoxyl, carbonyl, and carboxylic groups are the major functional groups present in lignin. Methoxyl group and β-O-4 were shown to be the dominant functional group and linkage, respectively in softwood and hardwood. Although ρ-hydroxyphenyl lignin (H-lignin), syringyl lignin (S-lignin), and guaiacyl lignin (G-lignin) are the primary monolignols for lignification in plants, recent studies have revealed that phenolic compounds other than these three monolignols occur in natural lignin. For example, catechyl lignin (C-lignin)*,* a new type of lignin, has been identified from the seed coats of several plant species including *Vanilla planifolia*, *Melocactus obtusipetalus*, *Ricinus communis*, *Mammillaria lasiacantha*, *Cleome hassleriana*, *Jatropha carcas*, *Aleurites moluccana* and *Vernicia fordii* (Chen et al., 2012; Chen F. et al., 2013; Tobimatsu et al., 2013; Zhuo et al., 2019; Su et al., 2021). Interestingly, C-lignin is a homopolymer with benzodioxanes as major linkages. Unlike H, G, and S type lignin, C-lignin is stable and does not become condensed during biomass pretreatment, thus it is considered as a perfect lignin for valorization (Li et al., 2018). Lignin plays a negative role in the lignocellulose conversion processes (Zoghlami and Paës, 2019), but on the other hand, lignin alone has also been considered an important fuel and a potential feedstock for aromatic products in biorefinery areas.

**FIGURE 1:**
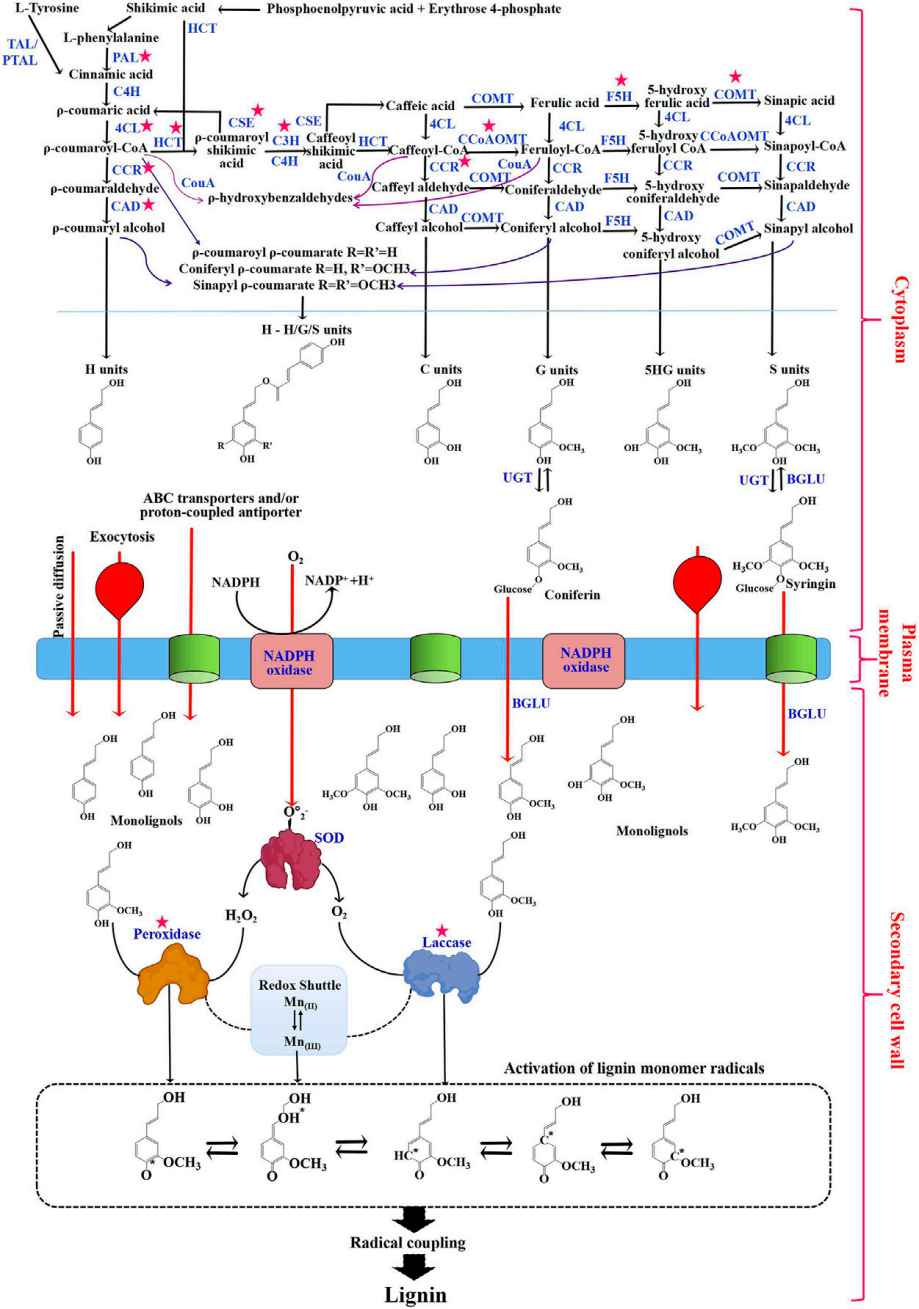
The general lignin biosynthesis pathway. Red arrows indicate the transport of monolignols from the cytoplasm through the plasma membrane into the secondary cell wall through passive diffusion/exocytosis/ATP-binding cassette (ABC) transporters. Dotted lines between the peroxidase/redox shuttle and laccase/redox shuttle indicate the regeneration of Mn [III] by laccase and peroxidase. Chemical structures in the dotted box represent activated monolignol radicals. Red stars indicate potential targets for plant genetic engineering to reduce lignin content and modulate the monomer composition of lignin. Abbreviations: TAL, tyrosine ammonia-lyase; PTAL, bifunctional phenylalanine and tyrosine ammonia-lyase; PAL, phenylalanine ammonia-lyase; C4H, cinnamate 4-hydroxylase; 4CL, p-coumarate: CoA ligase; HCT, hydroxycinnamoyl-CoA:CCR-cinnamoyl-CoA reductase; CAD, cinnamyl alcohol dehydrogenase; CSEvcaffeoyl shikimate esterase; C3H, ρ-coumarate 3-hydroxylase; COMT, caffeic acid O-methyltransferase; CCoAOMT, caffeoyl-CoA O-methyltransferase; F5H, ferulate 5-hydroxylase; CCR, cinnamoyl-CoA reductase; CouA, hydroxycinnamoylCoA hydratase/lyase; UGT, UDP-glucosyltransferase; BGLU, β-glucosidase and SOD, superoxide dismutase.

Lignin biosynthesis in plants has been widely studied and found to be conserved across species, being initiated from the phenylpropanoid biosynthesis pathway in plant cells. The phenylpropanoid biosynthesis pathway in most plants starts with the aromatic amino acid phenylalanine, or tyrosine in monocots (Barros et al., 2016; Rinaldi et al., 2016). Phenylalanine and tyrosine can be converted to cinnamic acid by phenylalanine ammonia-lyase (PAL), tyrosine ammonia-lyase (TAL), or bifunctional phenylalanine/tyrosine ammonia-lyase (PTAL) enzymes. Subsequently, cinnamate is converted to ρ-coumaric acid and then ρ-coumaric-CoA through cinnamate 4-hydroxylase (C4H) and 4-hydroxycinnamate CoA ligase (4CL) enzymes. In *Sorghum bicolor*, ρ-coumaroyl-CoA serves as a branching point for synthesizing flavones such as naringenin, apigenin, luteolin, chrysoeriol, selgin, tricetin, and tricin (Eudes et al., 2017). ρ-coumaric-CoA is on one hand converted to ρ-coumaryl alcohol (H-unit) by cinnamoyl CoA reductase (CCR) and cinnamyl alcohol dehydrogenase (CAD) enzymes (Chen H.-C. et al., 2013; Wang et al., 2013). On the other hand, it is used to produce caffeic acid and ferulic acid through a series of aromatic ring hydroxylation/methoxylation reactions by ρ-coumarate 3-hydroxylase (C3H)/cinnamate 4-hydroxylase (C4H), and caffeic acid/5-hydroxyconiferaldehyde 3/5-O-methyltransferase (COMT) enzymes. Then, caffeic acid and ferulic acid are converted to caffeoyl CoA and feruloyl CoA by 4CL and to caffealdehyde and coniferaldehyde by CCR, and finally to caffeyl alcohol (C-unit) and coniferyl alcohol (G-unit) by CAD. Sinapyl alcohol (S-unit) is synthesized using coniferaldehyde by ferulic acid/coniferaldehyde 5-hydroxylase (F5H), COMT, and CAD enzymes. S-unit can also be produced from coniferyl alcohol. After monolignols synthesis in the cytoplasm, glycosylated monolignols are then transported to the apoplastic space by passive diffusion, exocytosis, and ABC transporters (Wang et al., 2012; Figure 1). Lignin monomers can diffuse freely into the extracellular space but are only polymerized in the secondary cell walls, where they undergo single electron oxidation catalyzed by peroxidases and/or laccases. During the monolignols oxidation process using hydrogen peroxide or oxygen as the oxidant, phenoxy radicals were generated and then coupled to generate lignin polymers (Herrero et al., 2013; Lee et al., 2013; Zhao et al., 2013) (Figure 1). Several peroxidases have been identified from *Arabidopsis thaliana* (AtPrx 2, 4, 25, 37, 47, 52, 53, 64, 66, 71, and 72) (Herrero et al., 2013; Barros et al., 2015), *Zinnia elegans* (Novo-Uzal et al., 2013), and *Pyrus bretschneideri Rehd* etc., (Xie et al., 2021). A recent study has identified the potential role of peroxidases in lignification of Casparian strip in Arabidopsis (Rojas-Murcia et al., 2020). Similarly, recent studies have shown the involvement of laccases in lignification in several plants, such as *Arabidopsis thaliana* (LAC4, 11, 14, and 17) (Barros et al., 2015), *Setaria viridis* (SvLAC 9, 13, 15, 50, and 52) (Simoes et al., 2020), *walnut endocarp* (JrLAC12-1) (Li et al., 2021) and *Salvia miltiorrhiza* (SmLAC7 and 20) (Zhou et al., 2022). In *C. hassleriana*, laccase 8 (ChLAC8) has been shown to be involved in the polymerization of caffeyl alcohol to produce C-lignin (Wang et al., 2020).

This review briefly introduces the function and valorization of lignin from plants and summarizes the recent progress in lignin fractionation and depolymerization technologies. We discuss conventional strategies for genetic manipulation of lignin with core and non-core lignin biosynthetic enzymes and their side-effects. Then, we conclude how the emergence of spatio-temporal modification of lignin biosynthesis, mainly through specific promoters and post-transcriptional regulators, has shown promise for overcoming the adverse side-effects of conventional genetic manipulation methods. Finally, we provide a perspective on future research and exploitation of lignocellulose improvement lignin valorization through improved genetic manipulation strategies.

## Function and Valorization of Lignin

### Functional Significance of Lignin in Plants

As one of the essential components of the plant cell wall, lignin plays a vital role in plant growth and environmental adaptability. The primary function of lignin is providing mechanical strength to the cell walls in vascular plants. Numerous studies have shown that lignin deposition and lignification of plant cell walls is crucial for lodging resistance in rice, wheat, and other plant species (Zheng et al., 2017; Liu et al., 2018). Lodging resistance is one of the most important traits that affect crop growth and grain yield, as it can prevent plant stems from bending and breaking (Berry et al., 2004). The hydrophobicity and imperviousness of lignin also makes it important for the efficient transport of water and solutes through the vascular system (xylem and phloem). Besides the aforementioned functions, lignin can also protect plants from biotic (herbivorous insects, root-knot nematodes, phytopathogens) and abiotic stresses (conferring resistance against heavy metals, drought, salinity and temperature variations) by providing a physical barrier (Liu et al., 2018). Numerous lignin biosynthesis genes have been reported to play a role in response to various biotic or abiotic stresses. The differential lignification pattern has been detected in the roots and stems of *Leucaena leucocephala* developing under stress conditions, where lignin accumulation in developing stems was found to be significantly affected under drought and salinity stress conditions (Srivastava et al., 2015). Additionally, lignin is essential for seed production as lignin deposition in the seed coat provides mechanical protection from various stresses. For example, seeds with low lignin content from *ccr1* Arabidopsis mutant plants have reduced germination rate and modest growth (Vanholme et al., 2012). Similarly, mutations in the *C4H* gene involved in lignin biosynthesis affects the growth and male fertility in Arabidopsis (Schilmiller et al., 2009). Even though we have learned a great deal about the biosynthesis of lignin, the function of lignin and its related metabolisms in plants remains to be fully elucidated, which is important for the rational design of engineered lignin biosynthesis for improving lignocellulosic feedstocks.

### Valorization of Lignin Derived From Plant Biomass

Currently, the vast majority of lignin from crop production is burnt to generate heat and power, which not only contributed to environmental pollution but also wastes a versatile and valuable resource. Driven by the commercial availability of a large quantity of industrial lignin, its valorization to produce high-value bioproducts has been receiving increased attention in recent decades. Several techno-economic analyses have highlighted that lignin valorization is essential for the success of future lignocellulosic biorefineries (Ragauskas et al., 2014). In recent years, research on lignin valorization has mainly focused on producing aromatic building blocks, biopolymers, fuels, fatty acids, muconate, and lignin-based hydrogels/aerogels for drug encapsulation, tissue engineering or lignin-based nanoparticles/nanocomposites etc., For instance, using *Pseudomonas putida* KT2440, lignin derived aromatic compounds were transformed into muconic acids (4 g/L), which could be used for the synthesis of resins, food additives, plastics and fine chemicals of pharmaceutical (Salvachúa et al., 2018). In addition, lignin can be used as in antioxidants, sunscreen/ultraviolet (UV) rays protective agents, nucleating agents in thermoplastics and food packaging materials, flame retardant materials, surfactants, resins, and electrodes, among others (Lizundia et al., 2021). Biopolymeric food packing films with lignin have been shown to provide radical scavenging and UV protection (Mohammad et al., 2019). Additionally, blending native lignin, chemically modified lignin, nanolignin, and lignin-based nanofillers with flame retardant materials has significantly improved flame retardancy (Wu et al., 2021; Laoutid et al., 2022). Recent studies have demonstrated that jet fuels (precursors, hydrocarbons) can be produced from lignin through metal catalysts mediated depolymerization and hydrodeoxygenation (Chen et al., 2022; Kong et al., 2022). Despite these and other advances in converting lignin to chemicals, materials, and fuels, commercial applications are lagging behind fundamental research due to the limitations of fractionation and depolymerization technologies.

## Progress in Lignin Fractionation and Depolymerization

### Chemical Methods for Lignin Extraction From Biomass

The traditional approach to lignocellulose fractionation in the paper and pulping industries has generally focused on deriving cellulose or removing lignin using acidification, membrane filtration, and solvent extraction technologies, which usually lead to the degradation and condensation of lignin with a more complex chemical structure and lower reactivity for its conversion and high-value utilization. Optimal utilization of fractionated lignin from lignocellulosic biomass requires developing technologies that will maintain the original structure of lignin and efficiently separate it from lignocellulosic biomass. Fractionation of lignin and polysaccharides from lignocellulosic biomass depends heavily on different chemical pretreatments including acid pretreatment, alkaline pretreatment, steam pretreatment, and organic solvents pretreatment. For example, acid pretreatment can leave the majority of the cellulose and lignin in a highly digestible solid stream by efficiently solubilizing the hemicellulose. Thus, lignin is mainly recovered from the solid residue after enzymatic hydrolysis/fermentation. Conversely, lignin can be recovered from the liquid stream after being solubilized by alkaline pretreatment. Currently, solvent-based methods are used for solubilizing cellulose, hemicellulose, or lignin individually, or simultaneously from lignocellulosic biomass. Among them, organosolv methods provide high-quality, sulphur-free and solvent-soluble lignin that could be easily used for lignin valorization (Matsushita et al., 2015). The most commonly used solvents in the organosolv process are ethanol, methanol, acetic acid, formic acid, phenol, and acetone (Hallac and Ragauskas, 2014; Calvo-Flores et al., 2015; Moreno et al., 2017; Bajpai, 2018). Due to the flexibility of using different combinations of water, organic solvents, and catalysts, organosolv methods are attracting more research attention. The widely used organosolv processes are Alcell (Ethanol and water), acetocell (acetic acid and water), acetosolv (acetic acid and hydrochloric acid), ASAM (anthraquinone and methanol), Batelle/Geneva phenol (phenol, acid and water), Formacell (acetic acid, formic acid and water), Milox (formic acid and hydrogen peroxide) and organocell (Methanol, Sodium hydroxide and anthraquinone pulping) (Calvo-Flores et al., 2015; Bajpai, 2018).

Furthermore, combining solvent-based technologies with other methods may provide more advantages to existing lignocellulose fractionation facilities in biorefineries. This requires further research to develop strategies for isolating lignin from biomass using organic solvents, ionic liquids (ILs), and deep eutectic solvents (DESs) alone or in combination with thermo, mechanical and microwave-assisted methods (Zhou et al., 2022). The application of ILs for lignin fractionation from biomass has been gaining significant attention due to its nonvolatility, low melting temperatures, thermostability, eco-friendly nature, less impact on cellulose, and shorter reaction time (Rawat et al., 2022). ILs solubilize lignin from biomass by forming hydrogen bonds, covalent bonds, and pi stacking interactions (Singh, 2022), which has been reported as the ionoSolv process. For example, lignin isolated from *Eucalyptus nitens* wood using ILs such as 1-butyl-3-methylimidazolium hydrogen sulfate or triethylammonium hydrogen sulfate has less recalcitrance for further downstream processing (Penin et al., 2020). It is worth mentioning that ILs can depolymerize different types of lignin into valuable chemicals like vanillin, eugenol, guaiacol, diethyl maleate, phenol, and others, with high conversion rates and selectivity (Rawat et al., 2022). In addition, lignin can be selectively removed from biomass in its native form in high purity using pyridinium formate based protic ionic liquids (PILs) at 75°C (Hasanov et al., 2022). For example, poplar biomass pretreatment with PILs combined with green solvents such as gamma-valerolactone and levulinic acid has enhanced deligninfication, and thus preserves the structure of cellulose (Haykir, 2022). Furthermore, ILs, PILs, and DES can be combined with thermo-mechanical methods for biomass pretreatment. For example, pretreatment of mixed willow hardwood flour with 1-butyl-3-methylimidazolium chloride at 150°C for 45 min with high biomass loading yielded isolated lignin with high purity using a DES composed of choline chloride and lactic acid (Li et al., 2017; Guiao et al., 2022). Similarly using choline chloride and lactic acid-based DES, lignin (native form), cellulose, and xylose have been fractionated simultaneously from switchgrass lignocellulose (Chen et al., 2018). Using choline chloride and ethylene glycol-based DES, switchgrass has been delignified in a shorter time under optimal conditions without significant lignin condensation (Chen et al., 2020). In a recent study, Lou and Zhang (2022) reported a simple method for removing lignin with high yield and purity from wheat straw by presoaking in choline chloride and lactic acid-based DES at room temperature. Further development of economically feasible fractionation technologies is critical for the design of lignin-engineered plants and downstream processing technologies in the context of a biorefinery lignocellulosic biomass feedstocks.

Genetic manipulation of the lignin biosynthesis pathway in plants helps reduce lignin content, modification of composition or both, and maximizes lignin removal. Delignification of biomass from transgenic poplar trees with reduced CAD activity requires less alkali concentration for the kraft pulping process when compared to the wild type biomass (Pilate et al., 2002). Similarly, lignin could be extracted from transgenic poplar trees with high syringyl content and low syringyl content using a standard organosolv process (Luo and Abu-Omar, 2018). It is speculated that the biomass from transgenic plants/trees does not require significant changes in the existing lignin extraction methods. However, further studies are necessary at an industrial scale to confirm the feasibility of conventional and modern extraction methods to separate lignin from genetically modified plants.

### Microbial and Enzymatic Depolymerization of Lignin

Microbial/enzymatic depolymerization is an attractive and green way of producing commercially valuable chemicals from lignin. Several bacteria, fungi, and actinomycetes have been reported to depolymerize lignin by secreting multicopper oxidases (laccase), peroxidases, aryl alcohol oxidases, superoxide dismutases, cytochrome P450 s, and β-etherases (Xu et al., 2019; Zhu et al., 2022). The advantage of using enzymes for lignin depolymerization is the production of lignin monomers, dimers, and oligomers, which are further converted into valuable chemicals through chemical or biological transformation (Zhu et al., 2020; Mutanda et al., 2022). In addition, the presence of biological funnelling pathways in bacteria offers an eco-friendly opportunity to transform the lignin-derived aromatics into valuable chemicals like vanillin (Zhu et al., 2021). Interestingly, laccase depolymerized lignin has been transformed into lipids by Rhodococcus opacus (Wei et al., 2015). Co-cultivation of lignin depolymerizing Rhodococcus opacus PD630 and Rhodococcus jostii RHA1 VanA- yielded a higher lipid production than monoculture fermentation using lignin as a carbon source (He et al., 2017). Despite encouraging findings in microbial and enzymatic depolymerization of lignin, the identification of promising lignin degradation pathways and biological funneling pathways to produce the desired end products in microbes and their application in lignin valorization at the biorefinery scale is still in its infancy. There are still challenges to be overcome, such as the low yield of products, the toxicity of lignin depolymerization derived aromatics to microbes, inability to assimilate all lignin-derived aromatics of microbes, and lignin modifications during the extraction process. Complete depolymerization of lignin from lignocellulosic biomass using monoculture/genetically engineered microbes is time-consuming and exerts more metabolic and oxidative stress on microbes, thus remaining a difficult task (Zhu et al., 2018). Microorganisms usually coexist, communicate with each other, and drive various physiological/biochemical pathways through mutualism, synergism, and antagonism. Thus, a promising avenue to explore would be to construct synthetic microbial consortia through top-down, bottom-up approaches, and co-culturing techniques for optimal lignin depolymerization to produce desired end products with commercial importance at a large scale in a sustainable way. This would also distribute the metabolic labor among the members of the synthetic consortia. So far, wood-feeding nematodes, ruminants, compost soils etc., are considered as a potential source for constructing lignin-degrading microbial consortia (Lin, 2022). Recent developments in high throughput microbial community profiling and system biology approaches allow researchers to overcome the fragile nature of synthetic microbial consortia to harness its benefits for lignin depolymerization.

## Common Strategies for in Planta Lignin Modification

In the biorefinery utilization of plant lignocellulose, polysaccharide and lignin are the two main important polymers to be efficiently utilized. Due to the complex structure and composition of native lignin, efficient methods for obtaining pure products from lignin depolymerization are still a developing area. More attention has been given to transforming the polysaccharide components into bioethanol (Kriger et al., 2021). However, lignocellulosic polysaccharides are usually encrusted with lignin, which has long been considered an obstacle to the efficient degradation of polysaccharides (Zoghlami and Paës, 2019). Therefore, plant lignocellulosic materials with low lignin contents or easily removed lignin can help facilitate polysaccharide utilization. Genetic modification has proved to be a promising approach to artificially design biomass feedstocks with favorable properties. To alleviate the intractability of lignin, genetic reduction of lignin contents and/or altering lignin composition through classical forward genetic or targeted reverse genetic approaches are considered as one of the most effective strategies to improve the lignocellulosic biomass for bioenergy production (Pazhany and Henry, 2019).

### Genetic Manipulation of Lignin Biosynthetic Enzymes

Over the past 10 years, lignin biosynthesis pathways have been studied extensively and were found to be highly conserved across plant groups (Vanholme et al., 2019). These advances made it possible to take advantage of genetic modification technologies to modify lignin content and composition. Numerous genetically modified plants have been generated with downregulated expression of genes encoding the key lignin biosynthesis enzymes to improve the digestibility of lignocellulosic biomass (Poovaiah et al., 2014). To date, the genes and enzymes responsible for lignin biosynthesis have been identified in many model plant species, such as Arabidopsis, poplar, and rice. A total of 11 gene families including *PAL*, *C4H*, *4CL*, *HCT*, *C3H*, *CCoAOMT*, *CCR*, *F5H*, *COMT*, *CAD*, and *CSE*, are the most frequent targets of attempts to alter lignin (Figure 1). Some of them included numerous gene family members. The main findings of common genetic modifications related to the lignin biosynthesis enzymes are summarized in Table 1. Among them, *PAL*, *C4H*, *4CL*, *HCT*, *C3H*, and *CSE* generally participate in the initial stage of lignin biosynthesis, which is the general phenylpropanoid pathway (Jardim-Messeder et al., 2020). For example, the suppression of *PAL* genes in Brachypodium and tobacco generated mutant plants with reduced lignin levels and higher saccharification (Elkind et al., 1990; Sewalt et al., 1997; Cass et al., 2015). In Populus, genetic inhibition of *C4H* and *4CL* genes has also been shown to significantly decrease lignin content (Bjurhager et al., 2010; Voelker et al., 2011). Downregulation of other phenylpropanoid biosynthesis enzymes, such as *HCT*, *C3H*, and *CSE*, which work downstream of *4CL*, not only caused reduced lignin content, but also altered the accumulation of different monolignol units (Ralph et al., 2012; Ha et al., 2016; Ponniah et al., 2017). The rest of the enzymes, including *F5H*, *COMT*, *CCoAOMT*, *CCR*, and *CAD* participate in the monolignol branch pathways. It was reported that overexpression of *F5H* can increase the accumulation of S-lignin in tobacco, while suppression of *CCoAOMT* in *Pinus radiata* can not only reduce lignin content but also reduce G-lignin content and produce a new lignin constituent containing catechyl units (Franke et al., 2000; Wagner et al., 2011). In addition, downregulation of *CCR* and *CAD* resulted in reduced lignin content, and significantly increased bioethanol yield (Fornalé et al., 2012; Van et al., 2014). Notably, suppressing genes involved in the early steps of the phenylpropanoid pathway is the most effective way to reduce lignin content, while the downregulation of genes involved in the later branch pathway can significantly reduce the lignin S/G ratio but has little effect on lignin content. Taken together, targeting the genes involved in the lignin biosynthesis pathway has resulted in noteworthy progress in modifying the amount and composition of lignin in bioenergy crops.

**TABLE 1:** Common genetic modification of lignin biosynthesis in plants and its effects on plant growth and development.

Gene category	Gene name	Carrying promoter	Transgene techniques	Lignin content and H/S/G composition	Effects on plants	Host species and references
General phenylpropanoid pathway	*PvPAL ↓* ^a^	CaMV 35S	Sense-suppressed	Reduced and S/G ratio **↑**	Altered leaf shape, stunted growth, reduced pollen viability and changed flower morphology	*Nicotiana tabacum* Elkind et al. (1990); Sewalt et al. (1997)
	*BdPAL ↓*	Maize ubiquitin	RNAi	Reduced; S/G ratio and H units ↑	Delayed development and reduced root growth	*Brachypodium distachyon*; Cass et al. (2015)
	*LtC4H ↓*	CaMV 35S	Antisense-expressed	Reduced and S units ↑	Plants with distorted (curly) leaves, long internodes and thickened waxy leaves	*Lycopersicon esculentum*; Millar et al. (2007)
	*PtC4H ↓*	CaMV 35S	Antisense-expressed	Reduced and S/G ratio ↑	Impaired xylem conductivity and impeded water transport	*Populus tremula*; Voelker et al. (2011)
	*OsHCT* ↓	CaMV 35S	RNAi	Reduced and S units ↓	Normal phenotype and no lodging	*Oryza sativa*; Ponniah et al. (2017)
	*PgC3H ↓*	CaMV 35S	RNAi	Reduced and G units ↓, H units ↑	Normal growth phenotype	*Populus grandidentata*; Ralph et al. (2012)
	*MtCSE ↓*	N/A^b^	Mutant	Reduced and H units ↑	Severely dwarfed phenotype	*Medicago truncatula*; Ha et al. (2016)
Monolignol branch pathway	*AtF5H* ↑	CaMV 35S	Heterologous-overexpressed	No change and S units ↑	Not reported	*Nicotiana tabacum*; Franke et al. (2000)
	*PviCOMT ↓*	Maize ubiquitin	RNAi	Reduced and S/G ratio ↓	Normal growth phenotype	*Panicum virgatum*; Wu et al. (2019)
	*PrCCoAOMT↓*	Maize ubiquitin	RNAi	Reduced and G units ↓, H and C units ↑	Not reported	*Pinus radiata*; Wagner et al. (2011)
	*ZmCCR1 ↓*	CaMV 35S	RNAi	Reduced and not reported	The orange-brown colouration of the xylem	*Zea may*; Park et al. (2012)
	*ZmCAD2 ↓*	Maize ubiquitin	RNAi	Reduced and S/G ratio ↓	Normal growth phenotype	*Zea may*; Fornalé et al. (2012)
Lignin oxidation and polymerization	*Tp60 ↓*	CaMV 35S	Antisense-expressed	Reduced and S, G units ↓	Normal growth phenotype	*Nicotiana tabacum*; Blee et al. (2003)
	*AtLAC15 ↓*	N/A	Mutant	Reduced and not reported	Altered the seed coat color and slower root elongation	*Arabidopsis thaliana*; Liang et al. (2006)
	*BdLAC5 ↓*	N/A	Mutant	Reduced and S/G ratio ↓	Smaller stem height and internode diameter	*Brachypodium distachyon*; Cass et al. (2015)
TFs in lignin regulatory networks	*SbMyb60* ↑	E35S CaMV	Ectopic overexpression	Increased and S units↑	Ectopic lignification in leaf midribs	*Sorghum bicolor*; Scully et al. (2016)
	*PvMYB4* ↑	Maize ubiquitin	Ectopic overexpression	Reduced and S/G ratio ↑	Reduced plant stature and increased tillering	*Panicum virgatum*; Shen et al. (2012)
	OsMYB46 ↑; ZmMYB46 ↑	CaMV 35S	Heterologous-overexpressed	Reduced and not reported	Exhibit phenotypes of curly leaves and reduced rosette sizes	*Arabidopsis thaliana*; Zhong et al. (2011)
	*AtSND2/3/4/5* ↑	CaMV 35S	Ectopic overexpression	Reduced and not reported	Shorter stems with dark green curled leaves	*Arabidopsis thaliana*; Zhong et al. (2021)
	*PvKN1* ↑	CaMV 35S	Ectopic overexpression	Reduced and not reported	Shorter internodes and altered leaf structure including curly leaves	*Panicum virgatum*; Wuddineh et al. (2016)
	*SbbHLH1* ↑	CaMV 35S	Heterologous-overexpressed	Reduced and not reported	Stunted stem during reproductive growth	*Arabidopsis thaliana*; Yan et al. (2013)
	*VvWRKY2* ↑	CaMV 35S	Heterologous-overexpressed	Reduced and S/G ratio ↓	Delay in xylem formation	*Nicotiana tabacum*; Guillaumie et al. (2010)

a Arrows indicate up- or down-regulation of genes, and increases or decreases in lignin composition.

b N/A, not applicable.

### Genetic Manipulations of Non-core Enzymes and Regulators of Lignin Biosynthesis

Recently, plant peroxidases and laccases have attracted more attention for their involvement in the last step of monolignols oxidation and polymerization in lignin synthesis (Figure 1). Thus, manipulation of peroxidases and laccases has become an ideal strategy to modify the bioenergy plants to produce lignocellulosic biomass with better degradability. Downregulation of an anionic peroxidase in hybrid aspen has resulted into a decreased G-lignin content rather than S-lignin content, indicating a monomer-specific control of polymerization by peroxidases (Li et al., 2003). Interesting results have been obtained by overexpression of a putative lignoperoxidase in tobacco, which not only resulted in accumulated lignin, but also showed a protective effect against insect attacks (Dowd et al., 2000; Blee et al., 2003). Likewise, laccase gene families have been characterized in various plants, including Arabidopsis (17 isoforms, 6 subgroups), rice (30 isoforms, 5 subgroups), maize (5 subgroups), and Brachypodium (29 isoforms, 4 subgroups) (Caparrós-Ruiz et al., 2006; Turlapati et al., 2011; Wang, Y et al., 2015; Liu et al., 2017). Disruption of the gene encoding *LAC15* led to a reduced lignin content in Arabidopsis (Liang et al., 2006). The *BdLAC5*-misregulated mutant in Brachypodium has been reported to result in 10% decreased lignin content, change of the S/G ratio and higher saccharification efficiency (Cass et al., 2015). The results provide clear evidence that laccases are promising targets for alleviating the recalcitrance of lignin. Since the peroxidase and laccase gene families are relatively large, it still needs to be clarified whether there is redundancy or coordination among them during monolignol polymerization (during lignification).

With recent progress in understanding the molecular mechanisms on transcriptional regulation of lignin biosynthesis, an array of transcription factors that act as activators or repressors of the phenylpropanoid pathway and monolignols specific pathways were characterized in a broad range of plant species (Zhao and Dixon, 2011). New insights have been made towards targeting these different regulators to modify the lignification characteristics of plant biomass. Numerous transgenic and mutant plants have been generated with the manipulated expression of transcription factor genes under the control of the cauliflower mosaic virus 35S promoter (Table 1). Important examples include subgroup4 of R2R3-MYB transcription factors, which impart transactivation or transrepression of monolignol genes in the complex hierarchical transcriptional regulatory networks. For example, the overexpression of *SbMYB60*, a transcriptional activator regulating lignin biosynthesis, in sorghum resulted in a ∼10% increase in lignin content, leading to higher energy content of the biomass (Scully et al., 2016). While *PvMYB4* was identified as a transcriptional repressor of phenylpropanoid biosynthesis, its overexpression in switchgrass resulted in a ∼50% reduction in lignin and phenolic content, which in turn improved ethanol yields ∼2.5-fold (Shen et al., 2012). In another study, overexpression of *AmMYB308* and *AmMYB330* from Antirrhinum repressed phenolic acid metabolism and lignin biosynthesis in transgenic tobacco plants, which also repressed the activity of *4CL*, *CAD*, and *C4H* genes (Tamagnone et al., 1998). Other known transcription factors such as NAC, basic helix–loop–helix (bHLH), and WRKY have also been reported to be involved in monolignol biosynthesis. For instance, the overexpression of *SbbHLH1* in Arabidopsis resulted in significantly lower lignin content and a reduction in the expression of multiple lignin biosynthetic genes (Yan et al., 2013). Tobacco plants overexpressing *VvWRKY2* exhibited altered expression of genes involved in lignin biosynthesis pathway and showed a decrease in the lignin S/G ratio in both stem and petioles. The ability of *VvWRKY2* to activate the promoter of the *VvC4H* gene, was also confirmed by transient transcriptional activation assays in tobacco protoplasts (Guillaumie et al., 2010). Besides, MED5a/5b, the subunits of a large transcriptional co-regulator Mediator complex, have been reported to be involved in the repression of lignin biosynthesis in Arabidopsis, disruption of MED5a/5b in the Arabidopsis *ref8-1* mutant rescues the stunted growth, lignin deficiency and widespread changes in gene expression seen in the phenylpropanoid pathway mutant (Bonawitz et al., 2014). Overall, these transgenic results have proven informative to understand the regulatory roles of transcription factors (TFs) in lignin biosynthesis of lignocellulosic plants. Manipulation of these transcription factors can dramatically affect on lignin content and composition as these TFs can regulate several genes at once.

### Heterologous Expression of Microbial Ligninolytic Enzymes in Plants

With significant increase in the fundamental understanding of lignin degradation by fungal or bacterial depolymerizing enzymes, a variety of genes encoding ligninolytic degradation enzymes from microorganisms have been successfully expressed and targeted to subcellular compartments in different host plants (Table 2). For example, manganese peroxidase (MnP) from the white-rot fungus *Phanerochaete chrysosporium* that has been implicated in lignin degradation, were heterologously expressed in maize, which found to be enzymatically active and accumulates to high levels in transgenic maize seed (Clough et al., 2006). A fungal laccase gene *TvcvL3* identified from *Trametes versicolor* has been introduced into tobacco and rice, and a reduction of lignin content linked to the expression of fungal laccase were observed in transgenic rice lines (Furukawa et al., 2013). Feruloyl esterases (FAEs) have an important role in the enzymatic conversion of lignocellulosic biomass by decoupling plant cell wall polysaccharides and lignin. A type-A ferulic acid esterase (faeA) and a type-B ferulic acid esterase (faeB) from *Aspergillus niger* have been introduced to tall fescue and alfalfa plants, which exhibit modified cell wall morphology and composition with a reduction in ester linkages and elevated lignin content (Badhan et al., 2014; Buanafina et al., 2015). Besides, bacterial ligninolytic enzymes such as Ca-dehydrogenase (LigD, LigDFG) from the *Sphingobium*, DyP-type peroxidase (DypB) from *Rodococcus jostii* and hydroxycinnamoyl-CoA hydratase-lyase (HCHL) from *Pseudomonas fluorescens* have also been transformed in *Arabidopsis thaliana* or *Nicotiana benthamiana* host plants to mainly alter the lignin structure or produce new substructure lignin in a desired manner. Consequently, reduced lignin polymerization degree and improved saccharification efficiency could be observed in the transgenic plants (Eudes et al., 2012; Tsuji et al., 2015; Ligaba-Osena et al., 2017; Mnich et al., 2017). These results suggest that microbial genes are potential targets to engineer plants for better biomass processability that is otherwise difficult to achieve by using plant genes.

**TABLE 2:** Heterologous genetic modification of microbial ligninolytic enzymes in plants.

Gene category	Gene name	Carrying promoter	Transgene techniques	Lignin content and H/S/G composition	Effects on plants	Host species and references
Fungal source	*PcMnP* ↑^a^	CaMV 35S	Heterologous-overexpressed	N/A^b^	Abnormal leaf growth and morphology	*Zea mays*; Clough et al. (2006)
	*PcMnP* ↑	Seed-preferred promoter	Heterologous-overexpressed	N/A	Normal growth phenotype	*Zea mays*; Clough et al. (2006)
	*TvcvL3* ↑	CaMV 35S	Heterologous-overexpressed	Reduced and not reported	Not reported	*Oryza sativa*; Furukawa et al. (2013)
	AnfaeB ↑	CaMV 35S	Heterologous-overexpressed	Increased and not reported	Lower recalcitrance of transgenic lines	Medicago sativa; Badhan et al. (2014)
	*AnfaeA* ↑	CaMV 35S	Heterologous-overexpressed	Increased and not reported	Narrower shorted leaves, and decrease in biomass	*Festuca arundinacea*; Buanafina et al. (2015)
Bacterial source	*SpLigD* ↑	CaMV 35S	Heterologous-overexpressed	Increased G units	No visible differences in plant growth and development	*Arabidopsis thaliana*; Tsuji et al. (2015)
	*SpLigDFG* ↑	CaMV 35S	Heterologous-overexpressed	Increased S and G units	Not reported	*Arabidopsis thaliana*; Mnich et al. (2017)
	RjDypB ↑	CaMV 35S	Heterologous-overexpressed	No significant variation	Increased lignin degradation and improved saccharification	*Nicotiana benthamiana*; Ligaba-Osena et al. (2017)
	*PfHCHL* ↑	Secondary cell wall-specific promoter IRX5	Heterologous-overexpressed	No significant variation	Reduced lignin polymerization degree and improved saccharification	*Arabidopsis thaliana*; Eudes et al. (2012)

a Arrows indicate up- or down-regulation of genes.

b N/A, not applicable.

### Undesirable Side-effects of Conventional Lignin Modification

**FIGURE 2:**
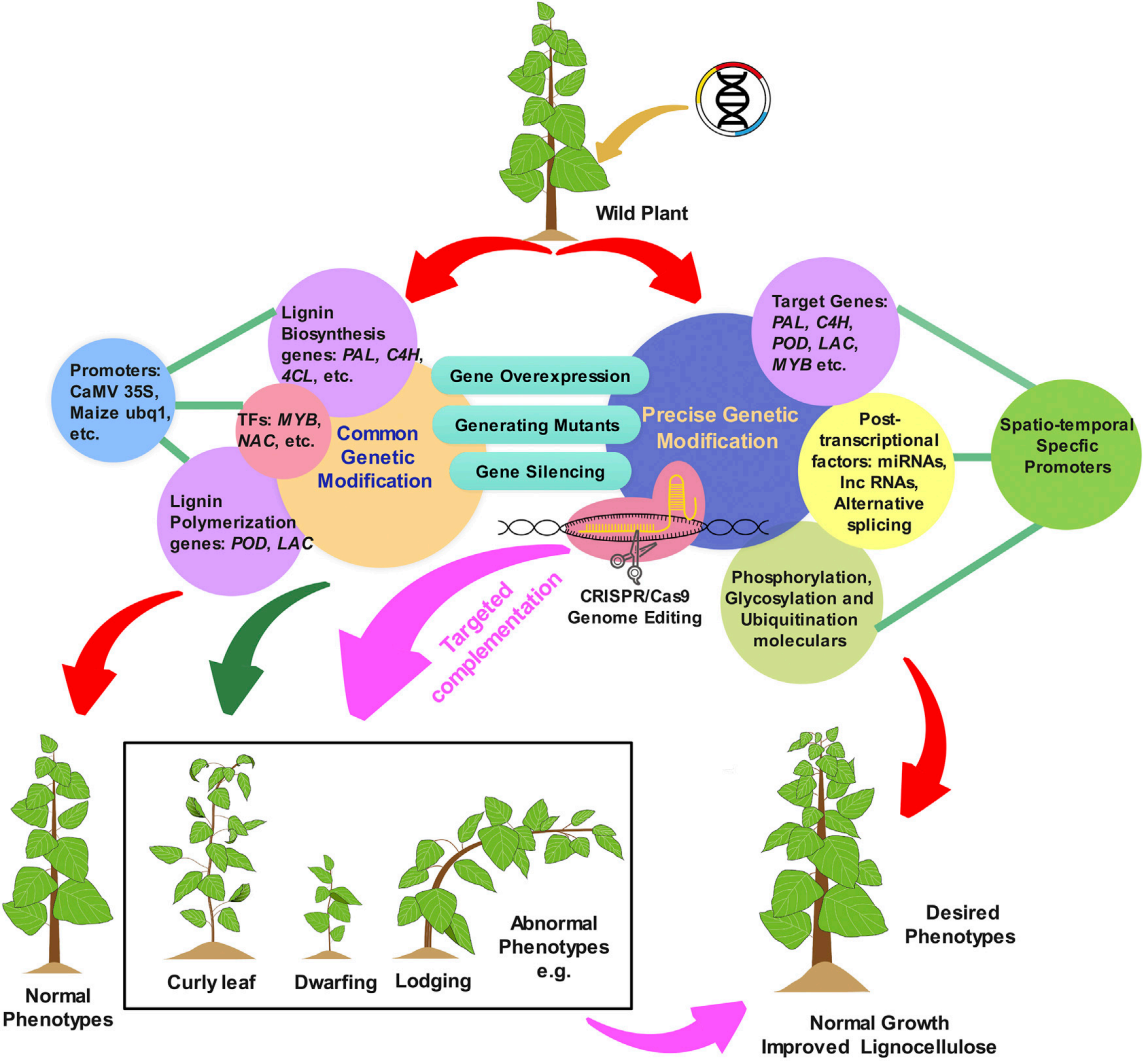
Schematic illustration showcasing strategies for lignin modifications in plants. Conventional approaches, including genetic modification of lignin through overexpression, silencing, or knockout of core or non-core lignin biosynthetic genes under constitutive promoters, often lead to undesirable phenotypes, such as lodging, dwarfing, and leaf curling. Precision genetic modification approaches utiliziong spatio-temporal specific promoters and post-transcriptional regulators have shown promise for overcoming the adverse side-effects of conventional approaches. CRISPR/Cas9 based genome editing technique are also anticipated to offer more flexibility in targeted engineering of lignification patterns in plants.

Great efforts have been invested in modifying lignin biosynthesis by manipulating key pathway genes or transcription factors to reduce lignin content and/or alter its composition. As discussed above, these conventional approaches can successfully improve lignocellulosic biomass digestibility. However, lignin-modification is a double-edged sword; while it can improve crop properties, it also often impairs plant growth and development, thus setting a limit on what is possible with conventional approaches (Figure 2). The most frequent abnormalities found in transgenic plants with significantly reduced lignin content are collapsed xylem, curly leaves, dwarfed stature, reduced fertility, decreased biomass production, water transport problems, and impaired agricultural fitness under biotic or abiotic stresses. For example, the downregulation of *CCR1* in maize results in abnormal plants with curly leaves and aborted early flowering (Park et al., 2012). Similarly, mutations of lignin biosynthetic genes such as *PAL*, *C4H*, and *CSE* in *Nicotiana tabacum*, *Lycopersicon esculentum*, and *Medicago truncatula*, respectively, resulted in abnormal phenotypes such as altered leaf shape (curly leaves), dwarfed growth, and changed flower morphology, etc, (Elkind et al., 1990; Sewalt et al., 1997; Millar et al., 2007; Ha et al., 2016). Severe downregulation of *4CL* in transgenic poplars with reduced lignin results in impaired xylem conductivity, which strongly impedes water transport (Voelker et al., 2011). Attempts to genetically alter lignin biosynthesis using transcription factors are also frequently accompanied by plant dwarfing or other developmental abnormalities. For example, transgenic switchgrass plants with an overexpression of *PvMYB4* had reduced lignin content and up to a 250% higher in biofuel production but at the same time were 40% shorter (Shen et al., 2012). Overexpression of *SNDs* genes, which belong to the MYB master switches known to regulate plant lignification and cell wall development, results in smaller rosettes with curly leaves in transgenic *Arabidopsis* (Zhong et al., 2011, 2021). These phenotypes could be the result of unforeseen metabolic plasticity, intrinsically variable transcriptional regulatory circuits, changes in spatio-temporal expression of genes, differences in cis-regulatory element composition of genes or protein-protein interactions controlling their distinct tissue organization and patterning, cell wall formation, and growth architecture. Therefore, developing optimized strategies to manipulate lignin biosynthesis without a growth penalty is crucial for overcoming the limits of lignin modification, and is the subject of the reminder of this review.

## Spatio-Temporal Patterns of Lignification

The spatial and temporal lignification patterns in plants are extremely important since it is a metabolically expensive process involving large quantities of carbon skeletons (Traversari et al., 2018). There is substantial diversity in the structure, content, and composition of lignins among plant species, tissue types, and growth stages of plants (Neutelings, 2011). For example, lignin staining of base internode sections of Brachypodium ecotypes using basic fuchsin and phloroglucinol exhibited a spatio-temporal patterning of lignification in grass stems, which showed a trend in the spread of lignification from the vasculature to interfascicular fibers throughout development (Kapp et al., 2015). This spread of lignification to non-vascular tissues may be advantageous for maintaining upright inflorescence stems for seed dispersal. In addition, lignification patterns in stems displayed spatial gradients within internodes. Similar patterns have been found in switchgrass stems, where acetyl bromide soluble lignin and phloroglucinol stained lignin increases from top to bottom of the second internode during stem elongation (Shen et al., 2009), and in sugarcane stems the lignification also starts at the early internode (top), and continues to the mature internode during the early developmental stage (Kasirajan et al., 2018). Besides, lignification patterns in leaves display a temporal parabola during maturation, such as in tobacco leaves, the accumulation of lignin gradually increased until reaching a peak at full maturity, and then gradually decreased, which is similar to the pattern observed in barley (Song et al., 2021).

Although molecular mechanisms underlying the spatio-temporal lignification patterns in plants have not been fully understood so far, several genes and regulatory factors mediating the dynamic control of lignification have been analyzed by global transcript profiling and other -omics approaches. A close spatio-temporal correlation between the lignification processes and gene expression in various plant tissues and growth stages has been established providing an indication of their probable role in the lignification patterns. Most lignin biosynthesis genes and their activators are expressed in the vascular bundles of the stems and leaves, particularly in the tissues undergoing lignification. For example, most lignin biosynthesis genes in flax are strongly expressed in the stems and the roots in the first 2–5 secondary xylem cell layers from the cambial zone towards the inner part of the organs. The expression intensity for these genes is generally higher in stems and roots than in leaves, except for *CCR* for which equal intensity was observed in all three organs (Leple et al., 2007). Furthermore, in *Eucalyptus gunnii*, *COMT* expression is present in the developing vessels of young stem internodes, while it was detected in all differentiating secondary xylem cell types in older stem internodes (Hawkins et al., 2003). In addition, many laccases and peroxidases are also expressed in different tissues at various developmental stages. For example, *AtLAC17* is mainly expressed in the interfascicular fibers, whereas *AtLAC4* is expressed in vascular bundles and interfascicular fibers (Lise et al., 2011). In turn, many lignin repressors are preferentially expressed in non-or poorly lignifying tissues, such as the shoot apical meristem. For instance, the transcription repressor *AtMYB32* is more highly expressed in flowers than in highly lignifying tissues, such as the stem, which explains the lower levels of lignin observed in floral tissues than in stems (Preston et al., 2004). Therefore, further exploring the correlation between the expression of lignin-related genes and the spatio-temporal lignification process is now of great interest for the optimized modification of lignin in transgenic plants. The extensive research on the spatio-temporal pattern of lignification and expression analyses of related genes has led to an improved understanding of lignin biosynthesis in plants. However, more information is needed to unveil the molecular mechanisms underlying these complex lignification process.

## New Insights Into the Precise Design of Lignin Modifications in Plants

Recent advances in understanding the complex regulatory system of transcriptional and post-transcriptional factors underpinning the spatio-temporal patterns of lignification in plants hold promise for designing novel protocols for lignin modification (Figure 2). Such experimental studies have greatly expanded the possibilities for the precise design of lignin modifications in a spatio-temporal manner to overcome some of the undesirable effects of downstream lignin modification. In the following paragraphs, we outline various strategies for targeted lignin modification to develop dedicated lignocellulosic feedstocks with desirable lignin traits as described in Figure 2. An ideal procedure would restrict lignin modifications to certain tissues or developmental stages by constructing the lignin related genes under the control of a tissue/stage-specific native or synthetic promoter. Additionally, using lignification associated post-transcriptional regulators such as microRNAs, alternative splicing isoforms and long non-coding RNAs to fine-tune constant lignin levels and compositions is another strategy to improve lignocellulose utilization. Lastly, sophisticated genome editing technologies such as clustered regularly interspaced short palindromic repeats (CRISPR) systems are also anticipated to offer more flexibility for engineering the lignin biosynthesis for targeted genetic modifications in plants.

### Promoter Panel for Spatio-Temporal Lignin Modification

The spatio-temporal patterns of lignification are usually associated with spatio-temporal expression of lignin biosynthesis and regulatory genes, which are precisely controlled by promoters. The most commonly used promoters to control gene expression in plant lignin modifications are constitutive promoters, such as the cauliflower mosaic virus (CaMV) 35S promoter (Zheng et al., 2007) and maize (*Zea mays*) ubiquitin 1 gene (Ubi-1) promoter (Rooke et al., 2000) (Table 1). Continuous and high-level gene expression driven by these promoters in all tissues, at all times, and under all conditions may not be necessary for crop improvement and could cause undesirable side effects, including suboptimal growth and epigenetic gene silencing (Figure 2). Thus, a wide variety of specific-expressed promoters have been intensively studied and utilized for plant genetic engineering in recent years, including plant native tissue-specific promoters, developmental stage-specific promoters, and artificially synthetic promoters. Unlike constitutive promoters, specific promoters drive transgene expression specifically in target tissues or certain developmental stages at manageable high levels. Using the β-glucuronidase (GUS) reporter system, promoters of several phenylpropanoid genes including *PAL*, *CAD*, and *CCR* were identified as strong vascular-specific promoters, which could drive the GUS gene expression exclusively in the vascular tissue in transgenic plants (Laible et al., 1997; Prashant et al., 2012). A similar result has been found on a phloem tissue-specific promoter PtrDP3, which could control GUS gene expression exclusively in phloem cells of the stem and root tissues of transgenic *Arabidopsis* and is suitable for phloem-specific biotechnological modifications in plants (Nguyen et al., 2017). In addition, the temporal pattern of *PtCOMT* promoter activity was previously found in the developing xylem of stems from the 8th to 13th week of the growth stage (Aronen et al., 2001). The specific regulatory roles of these promoters is mainly imposed by the presence of diverse, highly conserved cis-acting elements, which can be recognized and interacted by corresponding transcription factors to form a regulatory network. Promoter analysis and electrophoretic mobility shift assays have revealed that the secondary wall NAC binding elements (SNBE) corresponding to the NAC transcription factor-binding motif are required to specify the vessel-specific expression pattern of XCP1 gene in *Arabidopsis* and *Brachypodium* (Funk et al., 2002; Valdivia et al., 2013), and the AC elements (corresponding to the MYB transcription factor-binding motif) are necessary to regulate the tissue-specific gene expression of lignin biosynthesis (Zhao and Dixon., 2011). Identifying these critical cis-acting elements further enables the construction of synthetic promoters consisting of solely or multiple critical cis-acting elements and the basic promoter elements, which can also specifically regulate targeted gene expression.

Appropriate promoters enabling targeted expression of transgenes in particular cells, tissue types, or developmental stages have recently been utilized to efficiently obtain desired transgenic phenotypes (Table 3). Targeting lignin modification to specific plant tissue or stage can be achieved by constructing lignin biosynthesis or regulatory transgenes under the control of tissue- or stage-specific native or synthetic promoter. Research on this strategy of lignin modification can be categorized into two types from their experimental viewpoint. One approach is to target lignin biosynthesis repression through spatial restriction of the activity of a selected gene using suitable promoters. For instance, a *C4H* promoter in *Populus tomentosa* identified as temporally and spatially specific promoter has been fused to the antisense *CCoAOMT* cDNA for manipulating lignin biosynthesis, which suppressed lignin biosynthesis specifically in the vessel region can maintain growth while providing more sugar release in transgenic tobacco (Zhao et al., 2005). Although TFs have been proved to be powerful tools for modifying the lignin biosynthesis pathway, they could also be carefully regulated using tissue-specific promoters. Expected results have been obtained on the transgenic plants by direct use of a fiber-specific promoter from *PdDUF579-9* to drive a lignin-related transcription factor (*LTF1*), for modification of lignin biosynthesis in Populus, which restrict lignin suppression only in fibres and displayed vigorous growth with normal vessels (Gui et al., 2020). The second approach is to use appropriate specific promoters in a complementary manner to rescue the growth defects of critical tissues caused by constitutive knock-down or knock-out of lignin related genes (Figure 2). When using the vessel-specific promoter ProSNBE was used to drive the expression of *AtCCR1* in an Arabidopsis *ccr1* mutant background, the complemented *ccr1* ProSNBE:*AtCCR1* Arabidopsis plants showed partial restoration of lignification and cell-wall integrity in both vessels and neighboring xylary fibers, and thus restored plant biomass without influence the high saccharification efficiency of lignin mutants (De Meester et al., 2021). Collectively, these data highlight that specific promoters are more effective in precisely regulating and modulating lignin biosynthesis without compromising plant growth.

**TABLE 3:** Targeted genetic modification of lignin biosynthesis in plants using specific promoters and/or sophisticated genome editing technique.

Gene category	Gene name	Carrying promoter	Transgene techniques	Lignin content and H/S/G composition	Effects on plants	Host species and references
General phenylpropanoid pathway	*MsC4H ↓* ^a^	Vascular-specific promoter bean PAL2	Antisense-expressed	Reduced and S/G ratio ↓	Normal growth phenotype	*Medicago sativa*; Reddy et al. (2005)
	*AtC4H* ↑	Vessel-specific promoter VND6	Ectopic complementation of mutants	Reduced and not reported	Complete restoration of growth defect of the mutant	Arabidopsis *c4h-2*; Yang et al. (2013)
	*Pto4CL1* ↑	Xylem-specific promoter GRP1.8	Heterologous-overexpressed	Increased and not reported	Lignin content was increased in the stem, not in the leaves	*Nicotiana tabacum*; Lu et al. (2003)
	Pv4CL1 *↓*	Rice OsUbi2	CRISPR/Cas9	Reduced and S/G ratio ↑	Normal growth phenotype and improved sugar release	*Panicum virgatum*; Park et al. (2017)
	*AtHCT ↓*	Fiber-specific promoter, pNST3	CRISPR/Cas9	Reduced and not reported	Normal growth phenotype	*Arabidopsis thaliana*; Liang et al. (2019)
	*MsC3H ↓*	Vascular-specific promoter bean PAL2	Antisense-expressed	Reduced and S/G ratio ↑, H units ↑	Normal growth phenotype	*Medicago sativa*; Reddy et al. (2005)
	*AtCSE* ↑	Vessel-specific promoter VND7	Ectopic complementation of mutants	Increased and not reported	Restored the vasculature integrity resulting in improved growth parameters	Arabidopsis *cse-2*; Vargas et al. (2016)
	*PaCSE1 ↓*, *PaCSE2 ↓*	Arabidopsis U6	CRISPR/Cas9	Reduced and not reported	Normal growth phenotype and improved sugar release	*Populus alba*; Jang et al. (2021)
Monolignol branch pathway	*AtF5H* ↑	Lignifying cell-specific promoter C4H	Heterologous-overexpressed	Increased and S units ↑	Normal growth phenotype	*Nicotiana tabacum*; *Populus tremula*; Franke et al. (2000)
	*BnF5H ↓*	CaMV 35S	CRISPR/Cas9	Reduced and S/G ratio ↓	Improved disease resistance and increased stem strength	*Brassica napus*; Cao et al. (2022)
	*HvCOMT1 ↓*	wheat U6	CRISPR/Cas9	Reduced and S/G ratio ↓	Normal growth phenotype and improved sugar release	*Hordeum vulgare*; Lee et al. (2021)
	*PtoCCoAOMT ↓*	Lignifying cell-specific promoter C4H	Antisense-expressed	Reduced and not reported	Normal growth phenotype	*Nicotiana tabacum*; Zhao et al. (2005)
	*AtCCR1* ↑	Vascular-specific promoter bean ProSNBE	CRISPR/Cas9 complementation of mutants	Reduced and S/G ratio *↓* H units ↑	Normal growth phenotype and improved saccharification efficiency	Arabidopsis *ccr1*; De Meester et al. (2021)
Lignin oxidation and polymerization	*PrxA3a ↓*	Lignifying cell-specific promoter prxA3a	Antisense-expressed	Reduced and S units ↑	Normal growth phenotype	*Populus sieboldii*; Li et al. (2003)
	*PtoLAC14 ↓*	CaMV 35S	CRISPR/Cas9	Reduced and S units ↑	Normal growth phenotype with enhanced biomass saccharification	*Populus tomentosa*; (Qin et al., 2020)
TFs in lignin regulatory networks	*PdLTF1* ↑	Fibre-specific promoter PdDUF579-9	Ectopic overexpression	Reduced and S/G ratio ↓	Normal growth phenotype	*Populus deltoides*; Gui et al. (2020)
	*PtrMYB221* ↑	Xylem-specific promoter DX15	Ectopic overexpression	Reduced and not reported	Normal growth phenotype	*Populus tremula*; De Meester et al. (2021)
	*OsWRKY36 ↓*; *OsWRKY102 ↓*	CaMV 35S	CRISPR/Cas9	Increased and G units ↑	Altered culm morphology	*Oryza sativa*; Miyamoto et al. (2020)

a Arrows indicate up- or down-regulation of genes, and increases or decreases in lignin composition.

### Post-Transcriptional Regulation of Lignin Biosynthesis

Post-transcriptional regulation of mRNA or proteins exerts essential roles in many biological processes in plants (Guerra et al., 2015). Although much progress has been made on the transcriptional regulation of lignin biosynthesis and modifications, less is known about the post-translational regulatory mechanisms associated with lignification. The discovery of microRNA, alternative splicing, and long non-coding RNA provided preliminary evidence on the regulatory roles of mRNAs of genes related with lignin biosynthesis. At the same time, studies on phosphorylation, glycosylation, and ubiquitination increased the post-translational regulatory roles on the lignin biosynthesis related enzymes and proteins. Such regulation at the level of mRNA or protein offers more precise and quicker results in the modification of lignin.

MicroRNAs (miRNAs) are a class of small non-coding RNAs with a 21–23 ribonucleotide RNA sequence that play critical roles in gene expression regulation through directing mRNA cleavage or translational inhibition (Michlewski and Cáceres, 2019). MiRNAs have been found to regulate various developmental programs of plants, including the spatio-temporal lignification process (Rubinelli et al., 2013). Research in this area has already uncovered several beneficial modifications utilizing miRNAs useful for modulating lignin biosynthesis in transgenic plants. One such miRNA is *miR6443*, that is preferentially expressed in vascular tissues in *Populus tomentosa*, has been used to downregulate *F5H* expression in transgenic plants, which in turn resulted into a significant reduction in S-lignin content (Fan et al., 2020). In addition, *miRNA166* in *Acacia mangium* and *miRNA858a* in *Arabidopsis* were identified to have post-transcriptional roles in regulating TFs that control multiple phenylpropanoid pathway genes (Ong and Wickneswari., 2012; Sharma et al., 2020). Overexpression of these miRNAs resulted in the ectopic deposition of lignin in transgenic plants. One specific miRNA, *miRNA397*, which is highly conserved in several plant species, was consistently identified to regulate laccase genes and thus might be involved in monolignols polymerization (Sunkar and Zhu, 2004). One member of *Arabidopsis miRNA397*, *At-miRNA397b*, has been identified as a regulator of the *AtLAC4* gene, overexpression of which reduced lignin deposition through repression of the biosynthesis of both S- and G-lignin subunits (Wang et al., 2015). It is noteworthy that some of these miRNAs were identified to be tissue-specific, such as the *Populus tomentosa miR6443* and *Arabidopsis miR397b* were both found to be preferentially expressed in stem vascular tissues, and *Acacia mangium miRNA166* is differentially expressed between phloem and xylem (Lu et al., 2013). Thus, the spatial and temporal modifications of miRNAs will be needed to avoid side effects caused by the constitutive expression of miRNAs at a post-transcriptional level.

Recently, alternative splicing and long non-coding RNA of key regulators and enzymes have also been reported to play a critical role in the lignin biosynthesis pathway as a form of post-transcriptional regulation. Alternative splicing is an important modulator of gene expression that can increase the transcriptome plasticity and proteome diversity and thus contribute to the precise spatio-temporal transcript regulation (Laloum et al., 2018). Similarly, transcriptome analysis of 20 *Populus trichocarpa* genotypes has identified that about 40% of xylem genes are alternatively spliced, including lignin-related genes, such as *COMT*, *CCoAOMT*, *CAD*, and *C4H* (Bao et al., 2013). In addition, a *Populus* lignin-related TF, *PtrWND1B*/*PtrSND1*, was alternatively spliced *via* retention of intron 2 in the original transcripts, which led to the loss of DNA binding and transactivation activities and finally regulated the expression of the lignin-related gene *4CL1* (Li et al., 2012). Similar alternative splicing was also observed in its orthologs in *Eucalyptus*, but not in *Arabidopsis* (Zhao et al., 2014). Long non-coding RNAs (lncRNAs) are a class of transcripts with more than 200 nucleotides that lack a coding function (Kapranov et al., 2007). Genome-wide identification of lncRNA in *Populus tomentosa* has identified 16 genes targeted by lncRNAs involved in the lignification processes (Chen et al., 2016). In a similar study of cotton, a set of specific lncRNAs was enriched in lignin catabolic processes. These lncRNAs may regulate lignin biosynthesis by regulating the expression of *LAC4* (Wang et al., 2015). Although these studies imply the potential roles of alternative splicing and lncRNAs in lignin biosynthesis, the underlying regulatory mechanism and utilization in genetic modification of lignin remain unverified.

Moreover, the existence of several putative phosphorylation, glycosylation, and ubiquitination sites in sequences of critical lignin-related proteins added new clues to the post-transcriptional regulatory mechanisms for plant lignin biosynthesis. Protein phosphorylation is one of the most widespread post-translational mechanisms that regulate protein activity and stability. In *Populus trichocarpa,* phosphorylation was discovered to perform as an on/off switch of *PtrAldOMT2* activity in poplar monolignol biosynthesis (Wang et al., 2015). Besides, a lignin biosynthesis associated transcription factor (LTF) from *Populus*, *LTF1*, *via* down-regulating *4CL* to repress lignin biosynthesis, will be degraded through a proteasome pathway when it becomes phosphorylated (Gui et al., 2019). Glycosylation has been reported to be an essential regulation point in phenylpropanoid homeostasis for its direct roles on metabolites and monolignols (Le Roy et al., 2016). UDP-glycosyltransferases (UGTs) were suggested to be the main enzymes involved in regulating phenylpropanoid glycosylation status in different subcellular compartments. In *Arabidopsis*, members of the UDP-glycosyltransferase UGT72E and UGT72B subfamilies have been demonstrated to glycosylate monolignols (Speeckaert et al., 2020). The knockout of *UGT72B1* has resulted in extensively increased transcript levels of genes involved in lignin biosynthesis, polymerization and related transcription factors (Lin et al., 2016). Ubiquitination is a common regulatory mechanism in all eukaryotes that targets proteins for degradation. Kelch domain-containing F-box proteins (KFBs) are well known to specifically interact with PAL isozymes and mediate their ubiquitination and subsequent protein degradation (Kim et al., 2020). Double and triple mutants in Arabidopsis for the *KFB01*, *KFB20*, and *KFB50* genes showed an increased amount of *PAL* proteins, and consequently, more acetyl-bromide lignin in the plant cell walls, while overexpression of *KFBs* genes in transgenic plants caused a 2–70% lignin reduction (Zhang et al., 2013). To sum up, the discovery of phosphorylation, glycosylation and ubiquitination regulatory mechanisms on the lignification process has important implications for precisely modifying lignin biosynthesis, but more experimental work is required to support these hypotheses.

### CRISPR Technique for Defined Lignin Modifications

A predominant drawback of the antisense and RNAi system mostly used in the genetic modification of lignin is that the transgenic plants may not exhibit a stable phenotype, especially after several generations. Thus, using the sophisticated CRISPR/Cas9 (clustered regularly interspaced short palindromic repeats/CRISPR-associated protein 9) genome editing toolbox for the induction of desired traits/heritable mutations in a foreseeable genome location has received increasing attention (Scheben and Edwards 2018). The CRISPR/Cas9 system is used with a synthetic-guide RNA (sgRNA) containing ∼20 nucleotides complementary to the target locus, allowing targeted cleavage of genome DNA to knockout specific gene in an efficient and precise way. In the past several years, genome editing with CRISPR/Cas9 has been demonstrated in various plants, including biofuel species such as poplar and switchgrass (Table 3). For example, Jang et al. (2021) established a CRISPR/Cas9 system in poplar to target both the *CSE1* and *CSE2* genes related to lignin biosynthesis, and achieved reduced lignin content. Park et al. (2017) also employed the CRISPR/Cas9 system to knockout the *Pv4CL1* gene for the generation of switchgrass with low lignin content, and the resulted mutant switchgrass also exhibited an increased glucose and xylose release upon saccharification. Furthermore, a tissue-specific complementation strategy has also been established by combining a suitable tissue-specific promoter to maintain lignin reduction using the CRISPR/Cas9 system while minimizing the potential side effects (Figure 2). For example, an interfascicular fiber-specific promoter, pNST3, has been used to drive *CAS9* in the CRISPR/Cas9 system targeting *HCT*. Successful editing of the fiber-specific knockout of *HCT* was confirmed by observing mutations at the *HCT* target loci, and ∼90% decrease in HCT activity with a normal growth phenotype (Liang et al., 2019). Another artificial tissue-specific promoter, ProSNBE, has been used in the CRISPR/Cas9 system to target *CCR1* with modified codons to engineer plants with reduced lignin and normal growth phenotype with a single transformation event (De Meester et al., 2021). Although the CRISPR/Cas9-based genome editing technique is still in its infancies and with limited efficiency, it is anticipated to support precise editing and engineering the lignin biosynthesis in bioenergy plants.

## Summary and Future Perspective

Lignin is considered a vital component of lignocellulosic biomass that could potentially be converted into fuels and aromatic bioproducts. The structural complexity and diversity of lignin makes it challenging to extract from biomass selectively. Traditional separation methods generally focused on delivering high quality cellulose, while lignin was considered a waste product. Thus, most of the literature on lignin metabolic engineering focuses on reducing lignin contents to mitigate its intractability in biomass conversion (Table 1). With the intensive development of biorefineries for the production of fuels and chemicals from biomass, a biomass conversion strategy that maximizes the conversion of lignocellulosic biomass into high-value products, including readily fermentable sugars and lignin-derived chemicals (e.g., vanillin and lignosulfonates), has attracted more attention and has been considered in plant biomass upgrading strategies for economic and sustainable biorefineries. We reviewed the current research on the separation and extraction of lignin, both chemically (e.g., organic solvent, ionic liquids and low eutectic solvents methods) and biologically (e.g., microbial and enzymatic depolymerization). Developing new cost-effective strategies for lignin separation may provide novel opportunities for producing transgenic plants with increased lignin contents as an important breeding objective. Direct and constitutive genetic manipulations of lignin biosynthesis in plants, whether to increase or decrease lignin content, could lead to undesirable phenotypes that compromise the plants’ growth and development. Thus, defined strategies for spatio-temporal modification of lignin biosynthesis that we concluded in this review are prospected to produce biomass with elevated lignin production, together with more effective and efficient lignin conversion technologies, will promote the maximum utilization of lignocellulosic biomass.

In addition, genetic modification of lignin monomer compositions of subunits G, S, and H can also cause changes in lignin properties in modified plants, which offers another potential strategy to improve the convertibility of lignocellulosic feedstocks. The preferable lignin monomer composition depends on the intended use of the plant biomass. For the purpose of polysaccharides utilization, modification of lignin S/G ratio is one of the most common strategies of lignin engineering at the structural level used to reduce biomass recalcitrance and improve saccharification. S-lignin is less condensed compared to the H- and G-lignin monomers. The S unit proportion of lignin has been revealed to be the dominant factor that improves biomass digestibility, while the G-monomer reduces biomass digestibility. On the other hand, for the aromatics utilization purpose, simplifying lignin structures could contribute largely to produce higher yields and higher purity of aromatic products obtained following chemical or biochemical degradation of lignin. Pure G-lignin is already available as conifer lignin, which is simple in terms of aromatic ring composition. However, G lignin includes many substructures including degradation-resistant condensed substructures, which are rather recalcitrant to chemical utilization. H-lignin may have similar properties to G-lignin. Conversely, S-lignin consists mainly of b-O-4 substructures and has simpler structures than G or H lignin. In addition to the traditional H/G/S units of lignin, C-lignin made up of caffeyl alcohol exclusively was found to be synthesized in a spatially and/or temporally separated manner in seed coats of several dicot plants (Chen et al., 2012; Tobimatsu et al., 2013). It has been recognized as an ideal lignin feedstock which can lead to catechol derivatives through depolymerization (Berstis et al., 2016; Li et al., 2018). To date, successful cases regarding genetic modification of C-lignin biosynthesis in plants have not yet been reported. Further understanding of the mechanism of C-lignin biosynthesis will provide an alternative bioengineering approach to generate better biomass for biofuel production.

The extensive research on lignin biosynthesis pathways has made it possible to directly modify lignin contents and compositions in plants. The increasing progress in our understanding of the complex lignin regulatory networks, the transcriptional and post-transcriptional levels have greatly expanded the possibilities for the precise design of lignin modifications in a spatio-temporal manner to overcome some of the undesirable effects of lignin modification. Herein, we provide an update on cases where tissue/stage-specific promoters, post-transcriptional regulators, and sophisticated genome editing technologies have been used independently or in combination to precisely modify lignin content and compositions for *in planta* development of the lignocellulosic biomass leading to lesser undesirable traits and higher bioethanol yield (Figure 2). Furthermore, additional capable promoters with different tissue- or stage-specific regulatory roles should be explored from high-throughput genomic or transcriptomic databases. Subsequent functional characterization of isolated promoter sequences and critical cis-acting elements using various computational and experimental methodologies will largely expand the toolbox of available promoters for use in plant lignin manipulation studies. Finally, it would be informative to pay more attention to a full test of spatio-temporal growth and development alterations between lignin modified transgenic and wild plants, which is important for better understanding of the detailed effects of lignin modification on plants. Taken together, our review opens an avenue to more precisely manipulate lignin contents and compositions to improve lignocellulosic biomass properties and production.
